# Disproportionate Intrauterine Growth Intervention Trial At Term: DIGITAT

**DOI:** 10.1186/1471-2393-7-12

**Published:** 2007-07-10

**Authors:** Kim E Boers, Denise Bijlenga, Ben WJ Mol, Saskia LeCessie, Erwin Birnie, Marielle G van Pampus, Rob H Stigter, Kitty WM Bloemenkamp, Claudia A van Meir, Joris AM van der Post, Dick J Bekedam, Lucy SM Ribbert, Addie P Drogtrop, Paulien CM van der Salm, Anjoke JM Huisjes, Christine Willekes, Frans JME Roumen, Hubertina CJ Scheepers, Karin de Boer, Johannes J Duvekot, Jim G Thornton, Sicco A Scherjon

**Affiliations:** 1 Department of Obstetrics and Gynaecology, Leiden University Medical Center, The Netherlands; 2 Department of Social Medicine, Academic Medical Center Amsterdam, The Netherlands; 3 Department of Obstetrics and Gynaecology, Máxima Medical Center Veldhoven, The Netherlands; 4 Department of Medical Statistics and Bio-informatics, Leiden University Medical Center, Tthe Netherlands; 5 Department of Public Health Economy, Erasmus Medical Center Rotterdam, The Netherlands; 6 Department of Obstetrics and Gynaecology, University Medical Center Groningen, The Netherlands; 7 Department of Obstetrics and Gynaecology, Deventer Hospital, The Netherlands; 8 Department of Obstetrics and Gynaecology, Groene Hart Hospital Gouda, The Netherlands; 9 Department of Obstetrics and Gynaecology, Academic Medical Center Amsterdam, The Netherlands; 10 Department of Obstetrics and Gynaecology, OLVG Amsterdam, The Netherlands; 11 Department of Obstetrics and Gynaecology, St. Antonius Hospital Nieuwegein, The Netherlands; 12 Department of Obstetrics and Gynaecology, TweeSteden Hospital Tilburg, The Netherlands; 13 Department of Obstetrics and Gynaecology, Meander Medical Center Amersfoort, The Netherlands; 14 Department of Obstetrics and Gynaecology, Gelre Hospital Apeldoorn, The Netherlands; 15 Department of Obstetrics and Gynaecology, University Hospital Maastricht, The Netherlands; 16 Department of Obstetrics and Gynaecology, Atrium Medical Center Heerlen, The Netherlands; 17 University Medical Center St. Radboud Nijmegen, The Netherlands; 18 Rijnstate Hospital Arnhem, The Netherlands; 19 Department of Obstetrics and Gynaecology, Erasmus Medical Center Rotterdam, The Netherlands; 20 Department of Obstetrics and Gynaecology and Child Health, University of Nottingham, Nottingham City Hospital, UK

## Abstract

**Background:**

Around 80% of intrauterine growth restricted (IUGR) infants are born at term. They have an increase in perinatal mortality and morbidity including behavioral problems, minor developmental delay and spastic cerebral palsy. Management is controversial, in particular the decision whether to induce labour or await spontaneous delivery with strict fetal and maternal surveillance. We propose a randomised trial to compare effectiveness, costs and maternal quality of life for induction of labour versus expectant management in women with a suspected IUGR fetus at term.

**Methods/design:**

The proposed trial is a multi-centre randomised study in pregnant women who are suspected on clinical grounds of having an IUGR child at a gestational age between 36+0 and 41+0 weeks. After informed consent women will be randomly allocated to either induction of labour or expectant management with maternal and fetal monitoring. Randomisation will be web-based. The primary outcome measure will be a composite neonatal morbidity and mortality. Secondary outcomes will be severe maternal morbidity, maternal quality of life and costs. Moreover, we aim to assess neurodevelopmental and neurobehavioral outcome at two years as assessed by a postal enquiry (Child Behavioral Check List-CBCL and Ages and Stages Questionnaire-ASQ). Analysis will be by intention to treat. Quality of life analysis and a preference study will also be performed in the same study population. Health technology assessment with an economic analysis is part of this so called Digitat trial (Disproportionate Intrauterine Growth Intervention Trial At Term). The study aims to include 325 patients per arm.

**Discussion:**

This trial will provide evidence for which strategy is superior in terms of neonatal and maternal morbidity and mortality, costs and maternal quality of life aspects. This will be the first randomised trial for IUGR at term.

**Trial registration:**

Dutch Trial Register and ISRCTN-Register: ISRCTN10363217.

## Background

Around 80% of intrauterine growth restricted (IUGR) infants are born at term [1]. When pregnancy is complicated by IUGR, there is, whether term or preterm, a clear association with an increase in neonatal mortality and neonatal morbidity (short and long term) [2-4]. The long term morbidity ranges from behavioral problems and minor developmental delay to spastic cerebral palsy [5-10]. However, not all studies, especially after excluding congenital anomalies, confirm these findings [11]. Besides fetal asphyxia, meconium aspiration, fetal heart rate abnormalities and low Apgar score, also more admittances to and longer stays at neonatal intensive care units are reported. This might partly be related to a higher prevalence of hypoglycaemia, neonatal sepsis, hypothermia and haematological problems as thrombocytopenia and polycythemia in these neonates [12-14].

When a fetus is small for gestational age (SGA), defined on the basis of a birth weight below the 10^th ^centile, there is the concern that the fetus might be afflicted by IUGR [15]. As SGA is defined on the basis of an arbitrary chosen cutoff birth weight centile, not all infants falling below the 10^th ^centile are abnormally small because of growth restriction. Many neonates with a birth weight below the 10^th ^centile are representing the normal spectrum of fetal growth [11]. Variation in birth weight is related to many factors as maternal height, weight, parity and fetal gender, but also ethnicity [16]. For that reason optimal growth for any fetus should be related to the fetus' own individual optimal growth curve [17-19]. Intrauterine growth restriction has to be defined on further knowledge such as Doppler abnormalities as seen in placental perfusion, eventually in combination with abnormalities in cerebral perfusion [20,21] and possibly also by neonatal measurements as the Ponderal Index [22,23].

A reduction of fetal growth is exponentially associated with a higher perinatal mortality [24] and morbidity [25,26]. Doppler umbilical artery studies have shown that absence of end diastolic velocities, indicative of IUGR based on severe placental insufficiency is associated with a higher rate of caesarean deliveries and an increased incidence of perinatal and neonatal mortality [27-30]. However, a normal umbilical artery Doppler study at term gestation might be falsely reassuring, while a normal cerebral artery study might identify the fetus not likely having a major adverse outcome [31].

Most of the growth restricted children experience an accelerated growth, especially of the head circumference, during the first 6 months after birth [32]. However, this upward centile crossing or 'catch up growth' is not complete, even at the age of seven years [33]. Moreover head circumference seems to correlate with cognitive outcome [34].

Long-term neurological and cognitive development of the IUGR infant at term have been studied extensively. The Ponderal Index among IUGR infants, but also among infants with a normal birth weight, is an independent predictor of neonatal morbidity: the lower the Ponderal Index the higher morbidity [25]. Learning difficulties, defects in speech and mild neurological deficits and behavioral problems have been reported to occur more in term neonates born SGA [35,36]. At school ages (7–8 years) temperamental differences and differences in play behavior are apparent [37], most probably contributing to increased rate of school failure found in IUGR infants.

Long-term morbidity might be resulting from subtle nutritional insults to the brain in utero. Although the brain growth spurt, being the most vulnerable period of the human brain, spans a broad period between mid pregnancy and 6 months of postnatal age [38,39], it is shown that growth failure occurring around term shows a strong association with cognitive disturbances as a poorer mental and psychomotor development at two years of age [40]. However, not all studies, even at preschool age show this trend of increased problems in growth restricted infants [41,42]. Besides neurodevelopmental consequences it is now also clear that children who were undernourished during pregnancy (e.g. born with a birth weight more than 2 SD below the mean birth weight) and especially in combination having had a compensatory growth trajectory during childhood have an increased risk in later life for diabetes, hypertension and cardiovascular diseases [43].

Given the data from studies concerning the effect of under-nutrition on the brain and the effects on long-term cognitive and behavioral outcome, evaluation of the possible clinical benefit of early induction of delivery, pre-empting a detrimental effect of chronic under nutrition on the fetal brain intervention, is important. By such an intervention it might be possible to start earlier with a more optimal feeding, compensating for the poor intra-uterine environment. Induction of labor is very often common practice in cases of suspected IUGR [44,45]. In the Netherlands at 33 up to 36 weeks of gestation, 63% of IUGR pregnancies were induced, whereas from 37 weeks onwards this percentage is 23%; more than double the percentage in non-IUGR pregnancies. In a Dutch obstetric cohort of 14.294 primigravid women with IUGR pregnancies, 29% of these pregnancies were induced [46]. In these pregnancies complicated by IUGR, induction of labour was associated with an increased risk of instrumental deliveries and emergency caesarean section, but no difference in neonatal outcome immediately after birth was found.

At present, there is no uniformity on the management of women with IUGR at term. Although there is no doubt that the intra-uterine growth retardated fetus should be considered as high risk, and should be monitored, there is no consensus on which diagnostic methods to evaluate fetal condition and subsequent intervention is best. It is unclear whether in this situation either induction of labour or expectant management is beneficial for the mother and her baby, since evidence on the subject is lacking.

For preterm pregnancies complicated by intra-uterine growth retardation, an international randomised clinical trial recently showed that expectant management had little benefit over early delivery with respect to short term neonatal outcome [47]. However, results of this trial cannot be extrapolated to the situation at term.

The lack of consensus on the subject in the Netherlands is demonstrated by the fact that in 2002 in women with a SGA child, labour was induced in 32% of these women, whereas labour started spontaneously in 56% of these women, the remaining 11% had an elective caesarean section. These data are based on actual birth weight, and the clinical situation is even more complicated by the fact that the antenatal diagnosis of a SGA child is often difficult to make and easily missed in clinical practice.

In view of this clinical dilemma, we propose a randomised clinical trial in which induction of labour is compared with expectant monitoring in women with a suspected IUGR child at term. We will compare maternal outcome, neonatal outcome and maternal quality of life, as well as costs. Moreover, we will collect, in both randomisation arms, data of the diagnostic tests used in fetal surveillance, i.e. fetal heart rate pattern, sonographic measurement of the amniotic fluid index and Doppler measurement of the umbilical artery and the fetal medial cerebral artery in women.

## Methods/design

### Aims

The aim of this study is to investigate whether induction of labour or expectant management is the best strategy in terms of neonatal and maternal morbidity and mortality, costs and maternal quality of life aspect in pregnancies complicated by IUGR from 36 weeks gestational weeks onwards.

### Study design and setting

We will perform a randomised controlled multi centre study.

This trial is embedded in the Dutch Obstetric Consortium, a collaboration of obstetric hospitals in the Netherlands. Approximately 40 hospitals, including all 10 university hospitals, teaching hospitals and district hospitals will participate in this trial.

### Participants/eligibility criteria

All women with a singleton pregnancy, with a child in cephalic presentation, with suspicion of IUGR (Fetal Abdominal Circumference < 10^th ^centile, Estimated Fetal Weight < 10^th ^percentile as defined by local protocols), or decreased relative growth though still > 10^th ^centile, e.a. from 70^th ^centile to 40^th ^centile) are eligible. Gestational age should be between 36+0 weeks and 41+0 weeks. Women with a history of caesarean section, serious congenital defects, ruptured membranes, renal diseases, diabetes mellitus, or positive HIV serology will be excluded.

### Procedures, recruitment, randomisation and collection of baseline data

All women with a singleton pregnancy who present at one of the participating clinics will be referred to an obstetrician or a specifically appointed research nurse/midwife for counselling. Eligible women receive participant information. After written consent, they are randomised by means of a web-based application. Stratification will be applied for previous vaginal birth (nullipara versus multipara) and for centre. Randomisation will be in a 1:1 ratio for induction of labour or expectant management.

Patients that withhold consent for randomisation are asked permission for data collection on pregnancy outcome. Participation to the quality of life study and long-term follow up (Child Behavioural Check Lists-CBCL and Ages and Stages Questionnaire-ASQ) is asked separately.

Baseline demographic, past obstetric and medical histories will be recorded for all women. Cervical length will be measured at the time of randomisation. The quality of life questionnaires are filled out before randomization, after randomization, 6 weeks postpartum and 6 months postpartum. The questionnaires contain the Hospital Anxiety and Depression Scale (HADS), EuroQoL 5D3L, Short Form (SF-36), Symptom Check List (SCL-90), and questions on background characteristics, intervention preparedness, risk perception and experience with the current pregnancy.

### Intervention

When randomised to the induction arm, induction of labor must start within 48 hours after randomisation. Induction of labor can be proceded according to local protocol (among other things cervical ripening with prostaglandin-gel or tablets or with amniotomy, with or without the use of oxytocin). When allocated to the expectant management group patients will not be induced unless the fetal or maternal condition deteriorates and this is for the attending obstetrician a reason for induction. The patients will be observed, e.g. with fetal and maternal monitoring according to local practice, until labour starts spontaneously. However, monitoring must at least include measurement of the umbilical artery Doppler waveform, fetal heart rate tracing, blood pressure and urine analysis for albuminuria weekly. Doppler studies of the medial cerebral artery are optional. Reasons for interventions and time interval between randomisation and labour will be collected.

### Follow up of women and infants

All details of delivery, maternal and fetal assessments and admittance during pregnancy are recorded in the case record form that is accessible at the website. In case of admittance of the child to the neonatal intensive care unit, details of this admittance are also recorded.

Long-term follow up of children will be done by recording growth after birth as measured at the local infant follow up clinics.

### Outcome measures

The primary outcome measure will be a bad composite neonatal outcome. Adverse neonatal outcome will be defined as death before hospital discharge, a 5-minute Apgar score < 7, an umbilical artery pH < 7.05 or admission to the neonatal intensive care. Secondary outcome measures are mode of delivery and time until delivery, length of admittance at the neonatal intensive care, maternal morbidity, hospitalisation of the mother for fetal and maternal surveillance, quality of life, and costs. In the present proposal, no funding is asked for long term follow-up of the child, yet. However, if additional funding can be obtained children's behavioural-, and neuro development will assessed by administering with a postal enquiry the Child Behaviour Checklist-CBCL and Ages and Stages Questionnaire- ASQ by their parents after 2 years.

### Statistical issues

#### Sample size calculations

The study is designed as an equivalence study, whereby both treatments will have the same incidence of the primary outcome measure of combined bad neonatal outcome. This incidence is assumed to be 6% [46]. The null hypothesis is that both treatments will not be equivalent. To detect equivalence with a power of 80% a sample size in both groups of 325 will be needed (PASS SOFTWARE). The margin of equivalence, given in terms of the difference, extends from -5.5 % to +5.5 %. The actual difference is 0 %. The calculations assume that two, one-sided Z tests are used. The significance level of the test is 0.05.

#### Data analysis

Data will initially be analysed according to the intention to treat method. The main outcome variable, 'bad neonatal outcome', will be assessed by calculating rates in the two groups, relative risks and 95% confidence intervals as well as numbers needed to treat.

Time to delivery will be evaluated by Kaplan-Meier estimates, with account for differing durations of gestation at entry, and will be tested with the log rank test. The other secondary outcome measures will be approached similarly to the primary outcome measure. The analysis will be stratified for parity and centre.

#### Non response and inclusion bias

As non-response for follow up is overrepresented in certain outcome-related risk categories such as in non-native mothers, mothers with lower educational level and in mothers with boys, statistical methods that use imputation of missing data have to be applied [48]. To prevent inclusion bias all patients who were asked but decline randomisation, will be asked for permission to collect data on pregnancy outcome and further follow up according to the same schedule as the randomised patients.

### Economic evaluation

The aim of the economic evaluation is to compare optimality, in terms of costs and health effects, of both strategies. As the clinical study is based on equivalence design we hypothesize that there will be no relevant difference between maternal and neonatal outcome in the two strategies. The economic evaluation will be in the form of a cost-effectiveness analysis (CEA), in which the optimal strategy is defined as the strategy with the largest health gain at the smallest costs.

### Ethical considerations

This study has been approved by the ethics committee of the Leiden University Medical Centre (Ref. No. P04.210).

## Discussion

There is uncertainty about the management of IUGR at term, whether to leave the child in utero until spontaneous labour starts, or to prevent undernutrition by prolonged pregnancy in a poor intra-uterine environment by inducing labour. This latter treatment modality will most probably be at the cost of an increase in instrumental deliveries [46]. As optimal management of a pregnancy at term suspected to be complicated by IUGR remains unclear, it is a challenge to develop criteria for inducing delivery. An increase in fetal surveillance in these pregnancies (with normal umbilical artery studies) is thought to be associated with more inductions of labour and a shortening of gestational age [49]. Neonatal morbidity (and mortality) is low in term SGA neonates [3], nevertheless these neonates cannot be considered just "healthy small babies".

Although our primary aim is to study pregnancies complicated by IUGR, the inclusion criteria are obviously based on a suspicion of a SGA child, as we include women with a fetus with a Fetal Abdominal Circumference < 10^th ^centile or an Estimated Fetal Weight < 10^th ^centile. By patient's characteristics, such as ethnicity, maternal and paternal length as well as tests results as the amount of amniotic fluid or the Doppler of the arteria umblicalis, we will be able to evaluate which pregnancies are at risk for a poor neonatal outcome.

In summary, at the present, there is controversy as to which strategy is the best when IUGR at term is suspected. Whether to induce labour or to await spontaneous labour under strict fetal and maternal monitoring remains debatable because of a lack of evidence. Patients' management partly depends on the attending doctor and on local protocols. To resolve these issues, we will compare both strategies in the multi centre randomised trial – DIGITAT. In a pilot study carried out in one of the participating hospitals, we examined the feasibility of the DIGITAT-trial. Preliminary data from this small pilot show that the interval between randomisation and labour was 2 weeks shorter and birth-weight was 100 grams less in the pregnancies that were directly terminated by induction [50]. The results of the present DIGITAT-trial are expected in 2009.

## Abbreviations

IUGR – Intrauterine growth retardation

SGA – Small for gestational age

CBCL – Child Behavioural Check List

ASQ – Ages and Stages Questionnaire

NICU – Neonatal Intensive Care Unit

## Competing interests

The author(s) declare that they have no competing interests.

## Authors' contributions

SS, JvdP, BWM and JGT were involved in conception and design of the study. KEB and SS drafted the manuscript. All authors have read and given final approval of the final manuscript.

## Pre-publication history

The pre-publication history for this paper can be accessed here:


